# Pseudoexfoliation syndrome, a systemic disorder with ocular manifestations

**DOI:** 10.1186/1479-7364-6-22

**Published:** 2012-10-10

**Authors:** Eman Elhawy, Gautam Kamthan, Cecilia Q Dong, John Danias

**Affiliations:** 1 Department of Ophthalmology, SUNY Downstate Medical Center, 450 Clarkson Ave, Brooklyn, NY 11203, USA; 2 Department of Ophthalmology, Mount Sinai Medical Center, New York, NY 10029, USA

**Keywords:** Pseudoexfoliation syndrome, Glaucoma, Genetics, LOXL1, Exfoliation

## Abstract

Pseudoexfoliation syndrome (PXS) is a systemic condition with eye manifestations. In the eye, pseudoexfoliation material deposits on various structures of the anterior segment. The nature of this material is mostly fibrillar with fibers made up of microfibrils and coated with amorphous material. The composition of these fibrils is diverse and includes basement membrane components as well as enzymes involved in extracellular matrix maintenance. Pseudoexfoliation is the most common cause of secondary open-angle glaucoma (pseudoexfoliation glaucoma, PXG) worldwide. The goal of this review is to summarize our knowledge on the genetics of this systemic disorder and its resultant ocular manifestations. PXS familial aggregation suggests genetic inheritance. PXS has been strongly associated with single nucleotide polymorphisms (SNPs) of the lysyl oxidase-like 1 (LOXL1) gene on chromosome 15q24.1. Two of these SNPs confer a higher than 99% population attributable risk for PXS and PXG in the Nordic population; however, they carry different risks in different populations. The high risk haplotypes also vary among different populations. LOXL1 is one of group of the enzymes involved in the cross-linking of collagen and elastin in the extracellular matrix. Its function in connective tissue maintenance has been confirmed in mice; however, its actual role in PXS remains unclear. Contactin-associated protein-like 2 also has a strong genetic association with PXS in a German cohort and is an attractive candidate molecule. It encodes for a protein involved in potassium channel trafficking. Other candidate genes linked to PXS include lysosomal trafficking regulator, clusterin, adenosine receptors, matrix metalloproteinase-1 (MMP1), and glutathione transferase. These genes may be modifying genes for development of PXS and PXG.

## Definition

Pseudoexfoliation syndrome (PXS) is a complex systemic disorder of the extracellular matrix primarily affecting the eye and visceral organs [1]. Pseudoexfoliation material (PXM) deposits around blood vessels of connective tissue. It has been identified by electron microscopy [2,3] and immunohistochemistry [4] in the lung, liver, kidney, gall bladder, and cerebral meninges. Cardio and cerebrovascular disease such as angina, aortic aneurysm, and dementia have been linked to PXS [5-7]; however, this association remains controversial [8].

## Ocular manifestations

**Figure 1:**
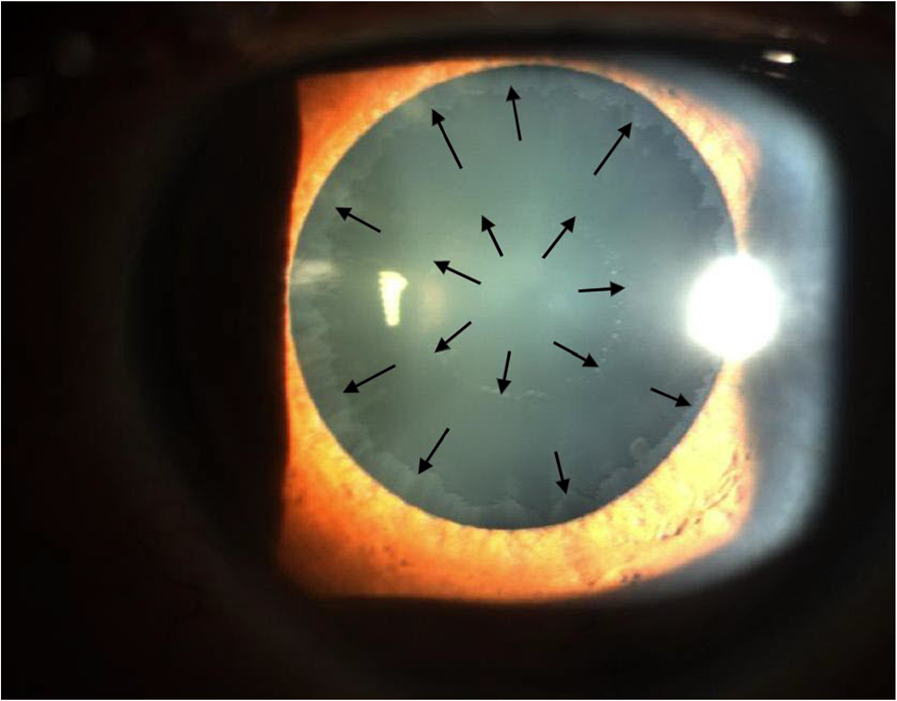
**Anterior segment photography of the eye following dilation of the pupil.** Notice the deposition of PXM on the anterior lens capsule. Arrows point to the characteristic double ring pattern.

**Figure 2:**
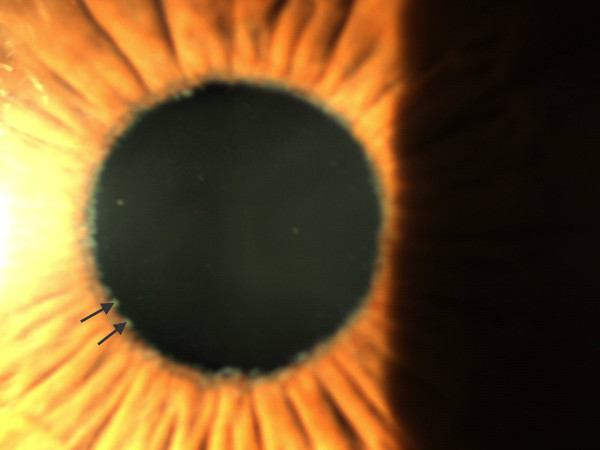
**Anterior segment photography of the eye showing PXM deposition at the pupillary margin (*arrows*).**

In the eye, pseudoexfoliation syndrome is characterized by the deposition of fibrillar material that can be found on all anterior segment structures bathed by aqueous humor. PXM can be observed *in vivo* during slit lamp examination. It appears as ‘dandruff-like’ material in the anterior chamber or most characteristically on the anterior lens capsule deposited in a double concentric ring pattern (Figure 1). The rings are separated by a clear zone presumably created because of the movement of the iris on the anterior lens surface. The central ring is located at the area of the iris sphincter, while the peripheral ring is only visible after pupil dilation. PXM is also often observed by slit lamp examination at the pupillary margin (Figure 2), on the lens zonules and on the trabecular meshwork. The site of production of this material which is a complex of various glycoproteins is unclear, but PXM can potentially originate from the iris, lens epithelium, ciliary body, or the trabecular meshwork [1].

Ocular manifestations of PXS include iris depigmentation leading to peripupillary transillumination defects, mild trabecular meshwork hyperpigmenation, secondary open-angle glaucoma, and phacodonesis or lens subluxation caused by zonular dehiscence. Loss of lens zonular support makes intraocular surgeries challenging with the potential for vitreous loss, lens subluxation, or even lens dislocation [9].

Although the ocular findings of PXS have been described more than 80 years ago [10], the exact pathophysiology of PXS is still obscure. Significant advances in our understanding, however, have been made in recent decades. This era of increased understanding starts with a hypothesis put forward by Barbara Streeten et al. [3], who suggested that PXS is a form of elastosis resulting from the overproduction of elastic microfibrillar components such as fibrillin-1 [11]. Further evidence that fibrillin overproduction or over aggregation may be causative for the formation of PXM came from the work of Schlotzer et al. who detected an increased extracellular deposition of fibrillin-containing fibrils in PXS [12].

## PXM pathology and nature

Transmission electron microscopy studies have confirmed that PXM is fibrillar in nature. Fibrils are composed of microfibrils, each 8 to 10 nm in diameter that undergoes lateral aggregation to produce complete PXM fibrils [11]. The PXM fibrils also have a coating of electron-dense amorphous material which often conceals the microfibrillar nature [11].

Immunohistochemical findings have suggested that PXM fibrils contain components of the elastic fiber and basement membrane system, such as elastin, tropoelastin, amyloid P, vitronectin, fibronectin, heparan sulfate proteoglycan, fibrillin-1, microfibril-associated glycoprotein, emilin, and latent transforming growth factor β binding proteins (LTBP-1 and LTBP-2) [11]. Liquid chromatography coupled with tandem mass spectrometry has confirmed the presence of fibrillin-1, fibulin-2, vitronectin, and amyloid P-component. In addition, it allowed the identification of the basement membrane components laminin, serum amyloid protein and fibronectin, desmosomal cadherins (desmocolin-2), the proteoglycans syndecan-3, and versican metalloproteases of the ‘A Disintegrin and Metalloprotease’ (ADAM) family (ADAMTS-8, 18, and 19), tissue inhibitors of metalloprotinases (TIMP3) the extracellular chaperone clusterin, and complement factor 1q (C1q) as components of PXM microfibrils [13].

It is unclear whether PXM accumulates due to excessive synthesis or inadequate breakdown. Elastic microfibril components, such as fibrillin-1, as well as other components of PXM such as LTBP-1 and LTBP-2 and the enzyme transglutaminase 2 were found to be upregulated at both the mRNA and protein levels in tissues of the anterior segment of the eye, where PXM can often be seen, suggesting excessive *de novo* synthesis as the cause [14]. However, an observed [15-17] imbalance between matrix metalloproteinases (MMPs) and their tissue inhibitors of metalloproteinases supports the possibility of improper PXM degradation. It is likely that PXM accumulation is a result of a combination of both excessive synthesis and insufficient degradation.

## Ocular complications of PXS

Pseudoexfoliation is the most common identifiable cause of open-angle glaucoma (OAG) [1]. In the Blue Mountain study (BMES) [18] performed on an Australian population of European origin, patients with PXS in either eye have a two to threefold higher risk of OAG, while eyes with PXS had fivefold increased risk for OAG even though OAG was often associated with only modest increase in intraocular pressure. The eye-specific in addition to patient-specific risk indicates the involvement of PXM in the development of OAG [18]. Retinal and optic nerve head pathology of pseudoexfoliation glaucoma (PXG) is considered to be similar if not identical to that of primary open-angle glaucoma (POAG). Axonal loss is seen in both; however, patients with PXG seem to have smaller decrease in capillary density compared with patients with POAG [19].

## PXS demographics

Pseudoexfoliation syndrome is a late onset condition with prevalence that increases markedly with age. In a Finnish population, its prevalence reached 33% among those aged 80 to 89 years. In Iceland the prevalence is 17.7% in patients aged 70 to 79 years and reached up to 40.6% in patients aged above 80 [20] years. PXS was also found to be generally more prevalent in men than in women [21]; this gender association though is not always reproducible [20]. The incidence of PXS roughly doubles each decade of life after the age of 50 [22] years. In Japan the incidence of PXS increased with age from 0.7% in ages 50 to 60 years to 7.3% in ages over 80 [22] years.

## PXS as a potential genetic disease

The association between human leukocyte antigen (HLA) and pseudoexfoliation suggests at least a genetic component to the inheritance of pseudoexfoliation syndrome [23].

The inheritance nature, however, has been difficult to determine because of late onset and the often asymptomatic nature of the process. Aggregation of the disease within families pointed to autosomal dominance inheritance [18]. Other inheritance patterns such as X-linked [24], maternal [25], and autosomal-recessive have also been suggested [26].

## PXS-associated genes and candidate molecules

Genome wide scan from one large pedigree suggested 18q12.1-21.33 as a promising locus. Other chromosomes that showed positive linkage were chromosomes 2, 17, and 19 but these overlapped with previously detected loci for POAG [27].

### Lysyl oxidase-like 1

A genome wide association study (GWAS) that was a part of the decode group, which included 90 cases of POAG, 75 cases of PXG, and 30 unclassified cases of Icelandic background, detected a strong association between PXG and a single nucleotide polymorphism (SNP), rs2165241 [28]. The SNP lies in the first intron of the lysyl oxidase-like 1 (LOXL1) gene on chromosome 15q24.1. The same group also pursued a confirmation of this finding with a larger GWAS study of 594 subjects affected with PXS or PXG and 14,672 controls from Iceland and Sweden. In the same chromosome, they uncovered two new SNPs rs3825942 (G153D) and rs1048661 (R141L) in the first exon of the LOXL1 gene, conferring a higher than 99% population attributable risk for PXS and PXG [28].

The three haplotypes (G-G, G-A, and T-G), formed by the alleles of the two nonsynonymous SNPs rs1048661 (R141L) and rs3825942 (G153D), carry different risks for the conditions with G-G and T-G being the high risk alleles while G-A being the low risk one [28]. T-A genotype was not observed in the study population.

Although the rs2165241 was more significantly associated with PXG than the two exonic SNPs, this association was no longer statistically significant after adjusting for the other SNPs at the same time [28].

The SNP rs2165241 located in the first intron of LOXL1 gene is strongly associated with PXG because it tags the G-G high risk allele for the SNPs rs3825942 and rs1048661 in the first exon of the LOXL1 gene [28].

In Iceland and Sweden the prevalence of the higher risk haplotypes is 50%, and thus these haplotypes are present in homozygous form in 25% of the general population over the age of 50 years. The risk of patients with this double homozygous genotype for developing PXS and PXG is thus 700 times more than those with only the lower risk haplotype [28].

The protein containing (Arg141-Gly153) is the one associated with the highest risk for PXG in Nordic and Caucasian populations. In contrast, the protein isoform (Leu141-Gly153) carries the lowest risk for PXG. However, this finding has not been replicated in other populations. Only the G allele of rs3825942 (G153D) is shown to carry high risk for PXS and PXG in both Caucasians and Japanese [29]. Although the high risk allele of rs1048661 is the G allele in Caucasians and south Indians [30], the T allele was the high risk allele in Japanese and Chinese [31,32]. In a black South African population, the G allele for rs1048661 (encoding arginine) was the same risk allele to that of Caucasians, while instead of the G allele the A allele of rs3825942 (encoding aspartic acid) was the other high risk allele [33].

It thus appears that because of their high prevalence in people without PXG in the Nordic population, the association of rs1048661 and rs3825942 SNPs with PXG may not be strong enough [34]. This difference in the high risk alleles in various populations also suggests the presence of other genes that contribute to the risk of PXG [33].

Nevertheless, LOXL1 remains an attractive candidate gene. LOXL1 is one of five enzymes in the family of lysyl oxidases, which are copper-dependent monoamine oxidases secreted by fibrogenic cells including fibroblasts and smooth muscle cells. The members of this family include lysyl oxidase (LOX) and lysyl oxidase-like (LOXL) 1, 2, 3, and 4 [35]. These enzymes are involved in the covalent cross-linking of collagen and elastin polymers in extracellular matrix formation. LOXL1 is necessary for tropoelastin cross-linking and elastic fiber formation, maintenance, and remodeling [36,37]. To accomplish this, the LOXL1 pro-peptide binds to both tropoelastin and fibulin-5 and selectively targets elastic microfibrils at the sites of elastogenesis [37]. Fibrillins and microfibril-associated glycoproteins are thought to form the scaffold to align cross-linking domains of tropoelastin [38]. After LOXL1 binds to the scaffolding, endo-metalloproteinase procollagen C-terminal proteinase (bone morphogenetic protein 1) cleaves the LOXL1 pro-peptide to prepare it for activation [36]. By oxidatively deaminating the lysine residues of tropoelastin, elastin fibers are covalently cross-linked.

The product of the LOXL1 gene thus modifies elastin fibers. The two single nucleotide polymorphism rs1048661 and rs3825942 associated with PXS translate into two different amino acids in the N-terminal of the LOXL1 pro-peptide at positions 141 (Arg instead of Leu) and 153 (Gly instead of Asp) [28].

Both coding SNPs in LOXL1 identified to date are in exon 1, which is known to encode a unique N-terminal domain of the LOXL1 proenzyme. Since this domain is significant for the correct enzyme activation and substrate recognition and binding, polymorphism in this region may affect enzyme function and subsequent PXM production [39].

Defective LOXL1 function has been studied in LOXL1 knockout (KO) mice where it results in multiple connective tissue defects. LOXL1 KO mice suffer from emphysematous changes in the lungs and vascular abnormalities resulting from a failure of elastic fiber maintenance [37]. Though LOXL1-deficient mice are viable and females are initially fertile, they undergo pelvic prolapse 1 to 2 days postpartum. The mice also experience increased laxity and redundancy of the skin, rectal prolapse, and intestinal diverticula. LOXL1 KO mice are prone to choroidal neovascularization after laser induction because of lack of elastic fiber maintenance in Bruch’s membrane [40].

These findings confirm the importance of LOXL1 in connective tissue maintenance. However, since PXM accumulation was not reported in these studies, it is unclear whether or not PXS occurs in LOXL1 KO mice. Even if absent in these animals, it is possible that a partial deficiency of LOXL1 may result in PXM formation, while complete loss of such activity may lead to minimal PXM formation [40].

### Lysosomal trafficking regulator

Animal studies have also suggested that the lysosomal trafficking regulator (LYST) gene is potentially important in PXS. LYST plays an important role in protein synthesis responsible for lysosomal function. Mutation of the LYST gene in human results in the lysosomal storage disease, Chediak-Higashi syndrome, which is characterized by immune deficiency, neurological manifestations, and bleeding tendency [41]. B6-*Lyst*^*bg-J*^ mice homozygous for the beige-J (bg-J) allele show multiple ocular features of human PXS [42]. Three key similarities were the pattern of iris transillumination defects caused by an unusual saw tooth-like morphology of the iris pigment epithelium, the accumulation of material resembling human PXM on the iris and elsewhere in the anterior chamber, and pronounced iris pigment dispersion. The beige mutation results from a 3-bp deletion causing the loss of a single isoleucine from the WD40 domain of the LYST protein, suggesting a disruption of protein-protein interactions. Though the LYST mutant mice do not resemble human PXS in all regards, they nonetheless have the potential to serve as an animal model for PXS, and may shed light on the complex genetic contribution and molecular pathway of the disease [42].

### Clusterin

Studies on clusterin have also indicated that its deficiency may result in PXM accumulation. Clusterin mRNA is expressed in most ocular cells and tissues, particularly in the epithelium of ciliary processes [14]; whereas the protein often localizes to extracellular structures, such as ocular basement membranes and stromal fibers. The presence of clusterin was also documented in the optic nerve and iris. In PXS eyes, a significant down regulation of clusterin mRNA was seen in all anterior segment tissues, irrespective of the presence of glaucoma, when compared to normal eyes and eyes with primary open-angle glaucoma [14]. Clusterin aqueous humor levels were also significantly reduced in PXS eyes [14]. *In vitro* analysis of nonpigmented ciliary epithelial cells has also shown significant down regulation of clusterin mRNA and protein upon exposure to transforming growth factor β1 (TGF-β1). TGF-β1 has been found to interact with lysyl oxidase in the formation of elastic fibers and is upregulated in anterior segment tissues in PXS (irrespective to the presence or absence of glaucoma) as well as in eyes with POAG. In addition to its interaction with clusterin and lysyl oxidase, TGF-β1 has been known to regulate other multiple factors involved in PXM accumulation, such as fibrillin-1, LTBP-1, LTBP-2, tropoelastin, and transglutaminase-2 [43].

The clusterin presence in pseudoexfoliation deposits and reduced amounts in aqueous humor of PXS eyes led to an investigation of the genetic variants of the clusterin (CLU) gene and its association with PXS. Nine SNPs across the CLU gene in 86 cases of PXS and 2,422 controls from the BMES cohort were genotyped. Variants of CLU gene do not strongly modify the risk of PXS in the Australian population, but one SNP (rs3087554) haplotype with 7% frequency may slightly increase the risk [44]. Due to the small size of the cohort and the significant age difference between cases and controls, the power of this study was low. Further studies are needed to verify any association [44].

### Adenosine receptor A3

Adenosine receptors are also candidate molecules. Adenosine is known to regulate aqueous humor secretion and affects intraocular pressure through its action on adenosine receptors. Differential expression of mRNA of adenosine receptors subtypes in PXS, PXG, and control eyes has been detected [45]. A3 receptor mRNA and protein were selectively upregulated and overexpressed in the basolateral infoldings of nonpigmented epithelium of the ciliary body in eyes with PXS with and without glaucoma. *In vitro*, A3 receptors can be selectively upregulated by hypoxia. PXS is characterized by anterior segment hypoxia [11] and this hypoxia-induced overexpression of adenosine receptors in PXS eyes not only indicates its cytoprotective role but also potentially suggests a new therapeutic option for PXS and PXG [45].

### Homocysteine metabolism genes

Another factor that may be associated with PXG is homocysteine. Increased plasma homocysteine level may be a risk factor for the development of glaucoma [46]. In a study of nonHispanic white population, plasma homocysteine levels were found to be increased in both PXS and PXG compared with controls [46]. Plasma homocysteine level can be elevated because of many factors such as genetic, folic acid deficiency, cobalamin deficiency, slow metabolism as in hypothyroidism, lack of excretion as in renal failure, and also aging. Methylenetetrahydrofolate reductase (MTHFR) controls homocysteine concentration and the mutation in the MTHFR gene can lead to reduced function and subsequent homocystinemia. Thus, MTHFR is another candidate gene [47]. The effects of polymorphisms in MTHFR and four other genes that are involved in homocysteine metabolism have thus been investigated. Those genes are methionine synthase (MTR), methionine synthase reductase (MTRR), methylenetetrahydrofolate dehydrogenase (MTHFD1), and cystathionine β-synthase (CBS). This study that only involved subjects of Caucasian European ancestry, however, failed to show a relationship of homocysteine levels with PXS or PXG and thus, homocysteine cannot be considered as a risk factor for PXS or PXG [48].

### Matrix metalloproteinases

Matrix metalloproteinases and their inhibitors are known to play a role in extracellular matrix maintenance and therefore may have an important role in PXS, either promoting or limiting the progression of the disease [3,49]. Based on studies of the genetic associations of matrix metalloproteinase-1 and 3 (MMP1) and (MMP3) gene polymorphisms with PXS in a Greek population, it has been suggested that there is a possible role for MMP1 variants in the development of PXS [49].

### Glutathione transferase

Glutathione transferase (GST) plays an important role in protecting cells from the oxidative damage caused by oxygen-free radical formed inside the cells. It conjugates those toxic products with glutathione, rendering them water soluble and excretable by the body [50,51]. It is found in the mitochondrial cytosole and lysosomes. The cytosolic form has three classes; mu (μ), theta (θ), and pi (π). The null genotype of mu is M0 and for theta is T0 [51]. Individuals carrying the null genotype have less protection against oxidative damage. Polymorphisms of GST have been detected in POAG [52], cataract [53], and exudative age-related macular degeneration [54]. However, in patients with PXG of Turkish [55], Swedish [56], and Arab [57] populations, no significant association was noted. Recently, it was shown that the M0 and T0 phenotype of GST is strongly associated with PXG in female Pakistani patients [56].

### Contactin-associated protein-like 2

A genome wide association study with DNA pooling also linked variants of contactin-associated protein-like 2 (CNTNAP2) gene on chromosome 7 (also known as Caspr2) to PXS [58]. Little information is available about the function of this protein. CNTNAP2 is a transmembrane scaffolding protein involved in the clustering of potassium voltage-gated channel subfamily A (K_*v*_1.1) [59]. It is believed to regulate potassium channels at neuron membranes and may have a role in membrane stabilization [60]. This gene has been implicated in several neuropsychiatric disorders [58]. To replicate the association findings, these SNPs were genotyped in an independent German cohort of 610 subjects with pseudoexfoliation syndrome/pseudoexfoliation glaucoma PEX/PXG and 364 controls as well as in 249 Italian subjects and 190 controls. A detailed analysis of this locus confirmed the association between two SNPs (rs2107856, rs2141388) and PXS/PXG in the German, but not in the Italian cohort. It also confirmed the two SNPs reported for the LOXL1 gene and detected an association of CNTNAP2 with the T (non-risk) haplotype of LOXL1 gene [58].

CNTNAP2 mRNA and protein distribution in ocular tissues were studied using real time p http://olymerase chain reaction and immunohistochemistry. CNTNAP2 localizes to epithelial and endothelial cells including the trabecular meshwork, Schlemm’s canal, cornea, nonpigmented ciliary epithelium, and even the retinal ganglion cells and optic nerve. The labeling of cell membranes is reduced in PXS and PXG patients [58].

### Tumor necrosis factor alpha gene polymorphism G-308A

Tumor necrosis factor (TNF)-α is a proinflamatory cytokine that has been implicated in various neurodegenerative disorders including glaucoma [61].

It is presumed to have dual roles depending on which receptor is activated. The activation of high affinity TNF-R2 receptor is thought to be neuroprotective, while activation of the low affinity TNF-R1 receptors mediates cell death through mitochondrial apoptosis [62]. An increased expression of TNF-α can shift the balance and stimulate TNF-R1 receptors [62].

TNF-α and its receptor (TNF-R1) m RNA were increased in the retina of glaucoma patients [63], while anti TNF-α antibodies can prevent ganglion cell death in a mouse model of glaucoma [64].

One SNP (G-308A of rs1800629) upstream of the promoter site of TNF-α activates the expression and causes elevation of TNF-α [62]. The association of this SNP with PXS has been recently studied in various populations. A strong association of GA and AA genotypes was observed with PXG in the Pakistani and Iranian populations [62,65]. However, this association was not confirmed in the Turkish [66] or Caucasian population [67].

### ABO blood groups

The association between blood groups and different glaucoma types was assessed in various case control and cross-sectional studies. A strong association between blood group type B and different types of glaucoma including PXG was found in Pakistani and Iranian populations [68,69]. The significance of this association is not clear at this time.

## Gene-environment interactions

Although familial aggregation, HLA and polymorphic marker association and geographical clustering suggest a genetic nature of PXS; environmental factors have been also suggested to play a role in the natural history of the disease [70].

The effect of ultraviolet (UV) light has been investigated, as PXS is more prevalent in populations with relatively high UV exposure [70,71].

Other environmental factors that have been proposed to participate in the pathogenesis of PXS are slow virus infection [72] and autoimmunity [73]. The report of the occurrence of PXS in relatively young patients after keratoplasty suggests the transmission of the disease from donor to the recipient. However, this cannot be considered proof, as development of PXS can also be the result of trauma or endothelial cell proliferation in the recipient eye [70,74]. Multiple case reports of young patients developing PXS after cataract surgery suggest that anterior segment trauma may be a predisposing factor in PXS development [75,76].

## Summary

Pseudoexfoliation syndrome is the systemic disorder with characteristic eye manifestations. It is the cause of one of the secondary open-angle glaucomas. Evidence suggests a strong genetic component to this condition (Table 1). To date a number of genes have been linked to PXS, of which LOXL1 appears to be the most relevant in many populations (Table 2). Recently, GST and CTNTAP2 genes were also found to be highly associated in some populations. Other candidate genes such as adenosine and clusterin, TNF-α, blood group type B show weaker associations but may contribute to the phenotype as modifying genes (Table 3).

**Table 1:** PXS-associated genes and associated polymorphic markers

**PXS associated gene**	**Chromosome**	**Associated polymorphic marker**
LOXL1 gene [28,29,32-34,77-80]	15q24.1	rs2165241
		rs3825942
		rs1048661
Clusterin gene [14]	8p21	rs3087554
Glutathione transferase gene [51,56,57]	1p13.3	M0 and T0 genotype
CNTNAP2 gene [58]	7q35	rs2107856
		rs2141388
TNF α G-308A polymorphism [62,65,67]	6p21	rs1800629
		rs361525
Blood group B [68]	9q34	

*Reported frequencies are for the rs1048661 T allele which is the high risk allele in the Japanese cohort.

**Table 2:** LOXL 1 SNPs high risk allele frequencies in different populations

**Population**	**Group**	**Cohort size**	**rs3825942 G**	**Odds ratio (OR)**	***P*value**	**rs1048661 G**	**Odds ratio (OR)**	***P *value**	**Reference**
			**Allele frequency**			**Allele frequency**			
Iceland	Control	14,474	0.847			0.651			[28]
	PXS	55	0.982	10.10 (4.02 – 25.36)	8.5 × 10^−7^	0.789	2.02 (1.32 – 3.09)	1.3 × 10^−3^	
	PXG	75	0.987	13.23 (5.59 – 31.29)	4. × 10^−9^	0.827	2.56 (1.74 – 3.77)	1.8 × 10^−6^	
Sweden	Control	198	0.879			68			[28]
	PXS	NA	NA			NA			
	PXG	199	0.995	27.28 (11.44 – 65.07)	9.1 × 10^−14^	0.834	2.39 (1.72 – 3.34)	2.7 × 10^−7^	
USA	Control	235	0.844			0.665			[34]
	PXS		NA			NA			
	PXG	50	0.939	3.05(1.20 – 7.76)	0.0194	0.787	1.86(1.10, 3.15)	0.0222	
India	Control	97	0.74			0.63			[30]
	PXS and PXG	52	0.92		0.0001	0.72		0.156	
Japan	Control	172	0.863			0.503*			
	PXS	103	0.985	10.71 (3.29 – 34.87)	1.49 *10^−7^	0.932*	13.56 (7.57 – 24.27)	3.39 *10^−28^	[29]
	PXG	106	0.986	11.02 (3.39 – 35.9)	1.40 *10^−7^	0.962*	25.21 (12.06 – 52.69)	1.44* 10^−34^	
Chinese	Control	171	0.918			0.444			
	PXS and PXG	62	0.992	1.92 (1.25 – 2.96)	0.0034	0.524	1.92 (1.25 – 2.96)	0.0034	[77]
German	Control	348	0.857			0.644			
	PXS	206	0.948	3.06 (1.87 – 4.99)	3.15 *10^−6^	0.787	2.04 (1.54 – 2.71)	7.08 * 10^−7^	[78]
	PXG	311	0.953	3.41 (2.22 – 5.24)	4.78 *10^−9^	0.839	2.89 (2.21 – 3.77)	1.40 *10^−15^	
Italian	Control	70	0.821			0.693			
	PXS	76	1.000	∞	5.08 *10^−8^	0.842	2.36 (1.34 – 4.16)	0.0024	[78]
	PXG	133	1.000	∞	1.96 *10^−12^	0.815	1.96 (1.22 – 3.15)	0.0053	
Australian	Control	86	0.84			0.66			
	PXS	335	0.95	3.81 (1.88 – 9.02)	7.83*10^−5^	0.78	1.86 (1.27 – 2.76)	8.49*10^−4^	[79]
	PXG		NA			NA			
Saudi	Control	101	0.817			0.762			[80]
	PXS	NA	NA			NA			
	PXG	93	0.968		0.000005	0.876		0.0056	

**Table 3:** Molecules related to PXS, function, and nature of association

**Molecules related to PXS**	**Known function**	**Nature of association**
Lysyl oxidase-like 1 (LOXL1) [28,32,34,78]	Copper-dependent monoamine oxidase secreted by fibrogenic cells. Catalyzes covalent cross-linking of collagen and elastin in ECM formation	-Gene polymorphisms linked to PXS in multiple studies
		-Protein present at site of pathology
Clusterin [14]	Clearance of cellular debris and apoptosis	-Clusterin deficiency associated with PXS.
		-Clusterin present in PXM deposits
Homocysteine and human cell metabolic enzymes (MTHFR MTR, MTRR, MTHFD1, CBS) [46,47]	Amino acids that participate in multiple metabolic processes.	Increased plasma levels associated with PXS
Glutathione transferase [50,51,56,57]	It conjugates those toxic products with glutathione, protecting cells from oxidative damage	-Linkage of null genotype of the GST gene with PXS
CNTNAP2 (Caspr 2) [58,60]	Regulation of potassium channels at neuron membranes. Possible role in membrane stabilization	-CNTNAP2 gene polymorphism associated with PXS
MMPs (MMP1) [15-17,49]	Extracellular matrix maintenance	-MMP1 gene polymorphism associated with PXS
Adenosine receptors [45]	Adenosine regulates aqueous humor secretion. Intraocular pressure are regulated through adenosine receptors	-A3 receptor mRNA and protein selectively up regulated in eyes with PXS
TNF-α [65]	Has dual action depending on the type of receptor activated.	-Increased expression of TNF-α shifts the balance and activates the low affinity TNF-R1 receptor leading to cell death
	High affinity TNF-R2 receptor has neuroprotective function while low affinity TNF-R1 receptors activation leads to cell death.	

## Competing interests

The authors declared that they have no competing interests.

## Authors' contributions

All authors read and approved the final manuscript.

## Grant support

This study was supported by NEI grants R01 EY15224, EY20670 and an unrestricted grant from Research to Prevent Blindness.
